# Full-Thickness Excision versus Shaving by Laparoscopy for Intestinal Deep Infiltrating Endometriosis: Rationale and Potential Treatment Options

**DOI:** 10.1155/2016/3617179

**Published:** 2016-08-04

**Authors:** Antonio Simone Laganà, Salvatore Giovanni Vitale, Maria Antonietta Trovato, Vittorio Italo Palmara, Agnese Maria Chiara Rapisarda, Roberta Granese, Emanuele Sturlese, Rosanna De Dominici, Stefano Alecci, Francesco Padula, Benito Chiofalo, Roberta Grasso, Pietro Cignini, Paolo D'Amico, Onofrio Triolo

**Affiliations:** ^1^Unit of Gynecology and Obstetrics, Department of Human Pathology in Adulthood and Childhood “G. Barresi”, University of Messina, 98125 Messina, Italy; ^2^Department of General Surgery and Medical Surgical Specialties, University of Catania, 95125 Catania, Italy; ^3^Department of Gynecologic Ultrasound Imaging, Altamedica Fetal Maternal Medical Centre, 00198 Rome, Italy

## Abstract

Endometriosis is defined as the presence of endometrial mucosa (glands and stroma) abnormally implanted in locations other than the uterine cavity. Deep infiltrating endometriosis (DIE) is considered the most aggressive presentation of the disease, penetrating more than 5 mm in affected tissues, and it is reported in approximately 20% of all women with endometriosis. DIE can cause a complete distortion of the pelvic anatomy and it mainly involves uterosacral ligaments, bladder, rectovaginal septum, rectum, and rectosigmoid colon. This review describes the state of the art in laparoscopic approach for DIE with a special interest in intestinal involvement, according to recent literature findings. Our attention has been focused particularly on full-thickness excision versus shaving technique in deep endometriosis intestinal involvement. Particularly, the aim of this paper is clarifying from the clinical and methodological points of view the best surgical treatment of deep intestinal endometriosis, since there is no standard of care in the literature and in different surgical settings. Indeed, this review tries to suggest when it is advisable to manage the full-thickness excision or the shaving technique, also analyzing perioperative management, main complications, and surgical outcomes.

## 1. Introduction

Endometriosis is a common benign and proliferative chronic disorder, characterized by the presence of endometrial glands and stroma outside the uterus. Ectopic endometrial tissue shows the same cyclic changes of the eutopic endometrium, according to the various phases of the menstrual cycle. The incidence in the female population is about 6–10%, with an average age at diagnosis ranging from 25 to 30 years [1, 2]. Endometriosis most frequently occurs in the pelvis. Therefore, its most distinctive presenting clinical features are menstrual irregularities, chronic pelvic pain, dysmenorrhea, dyspareunia, and infertility. The natural history of the disease has never been well defined because a consistent part of affected women are asymptomatic. Endometriosis is often diagnosed during laparoscopic investigation due to infertility [3, 4]. Three main clinical presentations have been described: peritoneal endometriosis, endometriotic ovarian cysts (i.e., endometriomas), and deeply infiltrating endometriosis (DIE) [5]. The latter is considered the most aggressive presentation of endometriosis, penetrating more than 5 mm in affected tissues [6] and affecting approximately 20% of all women with the disease [7, 8]. Endometriosis affects the bowel in 3%–37% of all cases, and histopathological diagnosis is usually straightforward [9]. More than 80% of digestive localizations concern the rectum and the distal sigmoid colon, and those lesions appear as fibrotic nodules also infiltrating the vagina, the uterine isthmus, the uterosacral ligaments, or the adnexa. Intestinal DIE is often associated with ovaries and ureters coinvolvement, showing the most aggressive presentation [10]. DIE can cause a complete distortion of the pelvic anatomy, and it mainly involves uterosacral ligaments, bladder, rectovaginal septum, rectum, and rectosigmoid colon [11–13]. These infiltrating lesions respond as other implants to various hormonal therapies, but it is not a definitive management for symptomatic patients, for which a surgical treatment may be required [14]. Multiple minimally invasive surgical approaches and techniques are available for treatment of intestinal endometriosis and often require the expertise of both gynecologist and general or colorectal surgeons. The purpose of endometriosis surgery is to obtain good long-term outcomes regarding pain relief, recurrence rates, and fertility and to not compromise the function of involved organs. The laparoscopic shave excision consists in dissection, maintained as superficial as possible, to avoid compromising bowel integrity. Depending on the depth of lesion, to diminish the risk of postoperative bowel perforation, laparoscopically placed sutures are required if a portion of the intestinal muscularis propria is dissected. Intraoperative visual inspection with proctoscopy and an air leak test can ensure that no inadvertent proctotomy exists [15]. For DIE nodules of the rectum, the rectal shaving can be performed using traditional shaving technique, releasing first the nodule from the rectal wall, or with reverse technique, starting the resection from the posterior vaginal fornix [16]. Mucosal skinning consists of removing the DIE nodule from the bowel deep in the layers of the intestinal wall, keeping just the mucosa intact. The defect in the rectal wall is sutured at the end of the procedure [17]. Full thickness or disc excision is performed using electrocautery or carbon dioxide (CO_2_) laser to perform the complete excision of nodules, after adequate laparoscopic mobilization of the intestine. The bowel is then repaired by laparoscopic suturing in the transverse axial plane to avoid potential stricture of the bowel lumen; alternatively an endolinear stapling device can be used [18]. In the anterior rectal wall, endometriosis nodules that are less than 3 centimetres in diameter and occupy less than one-third of the circumference can be treated with an alternative “closed” approach using a circular stapler, introduced transanally, that removes a full-thickness patch of the anterior rectal wall. The main advantage of this technique is a reduction of postoperative infectious complications [19, 20]. Laparoscopic resection of any gastrointestinal tract segment can be performed using more than one potentially successful strategy.

This review describes the state of the art in laparoscopic approach for intestinal DIE with a special interest in intestinal involvement, according to recent literature findings. Our attention has been focused particularly on full-thickness excision versus shaving technique in DIE with intestinal involvement. Particularly, the aim of this paper is clarifying from the clinical and methodological points of view the best surgical treatment of deep intestinal endometriosis, since no standard of care exists in the literature and in different surgical settings. Indeed, this review tries to suggest when it is advisable to manage the full-thickness excision or the shaving technique, analyzing also the perioperative management, the main complications, and the surgical outcomes.

## 2. Materials and Methods

This paper is a narrative overview synthesizing the findings of literature retrieved from searches of computerized databases. The database PubMed (National Center for Biotechnology Information, US National Library of Medicine, Bethesda, MD, USA) was used. Key research words were “endometriosis,” “deep endometriosis lesions,” “intestinal deep endometriosis involvement,” “rectovaginal endometriosis,” “laparoscopy in endometriosis,” and “surgical technique of endometriosis.” We focus our discussion on two different surgical laparoscopic techniques: full-thickness excision versus shaving. We looked for all original articles published in English through the end of 2014 and decided to extract every notable item of information concerning definition, symptoms, clinical features, differential diagnosis, preoperative evaluation (e.g., ultrasonography, MRI, rectal sigmoidoscopy, or colonoscopy), type of medical and surgical approach, type of complications, and postoperative approach in intestinal DIE.

## 3. Results and Discussion

### 3.1. Histology

Bowel endometriosis typically involves the serosa and muscularis propria, rarely involving the submucosa or mucosa, and usually is situated in the antimesenteric edge of the bowel [10, 21] and differs from peritoneal and ovarian implants, since they consist of smooth muscle with active glandular epithelium and scanty stroma. Mural thickening and intestinal stenosis are produced by fibrosis when larger endometriotic nodules invade the muscularis [22, 23]. There are two important basic characteristics of bowel endometriosis: multifocality and multicentricity. The former is defined as the presence of other lesions within a 2 cm area from the main lesion, and the latter is defined as the presence of other lesions beyond 2 cm from the main lesion. They seem to occur in 62% and 38% of surgical specimens, respectively [24, 25].

### 3.2. Clinical Presentation

The complexity of endometriosis results from its multiple clinical presentations, the multifocal pattern of distribution of the lesions, and the difficulty in the preoperative diagnosis [17]. The natural history of the disease has never been well defined due to its asymptomatic nature in a quite large number of the cases. In the mid-1990s, only 50% of deep endometriotic nodules >3 cm in diameter were diagnosed by physical examination [26]. With experience and awareness on the part of practitioners, the clinical diagnosis has improved. Nevertheless the use of only a physical examination continues to misdiagnose the vast majority of DIE. In women with moderate-to-severe presentation of the disease, some degree of intestinal symptoms may be present. Endometriosis-related intestinal symptoms may vary depending on the site of endometriotic implants and menstrual cycle [27]. It should be suspected in all women with invalidating hypogastric pain, especially dysmenorrhea, deep dyspareunia, severe chronic pain, mictalgia, and dyschezia. Most pathognomonic signs are severe dyschezia, menstrual blood on stools, menstrual diarrhea, severe menstrual mictalgia, and radiation of pain to the perineum [11]. Symptoms can be nonspecific with considerable overlap with other clinical conditions, delaying diagnosis and treatment. Moreover, physical examination (especially vaginal examination) may be completely normal, which hampers the diagnosis in young females. Chronic pelvic pain, often more severe during menstruation or ovulation, is the most common symptom associated with endometriosis. Rectal involvement may result in alterations in bowel habits such as constipation, diarrhea, dyschezia, tenesmus, and, rarely, rectal bleeding [28–30]. Intestinal perforation due to endometriosis may occur in the colon [31] and also in an appendix with transmural endometriosis. Typical endometriosis symptoms, however, also occur in patients with other conditions such as irritable bowel syndrome (IBS) and pelvic inflammatory disease (PID). These overlapping symptoms create potential diagnostic difficulties because there are no simple noninvasive diagnostic tests that can be carried out. Differential diagnosis includes irritable bowel syndrome, solitary rectal ulcer syndrome, and rectal tumor [28]. Colonic endometriosis must be differentiated from Crohn's disease, diverticular disease, adhesions, or neoplasm. Also, for small bowel implants secondary to endometriosis, difficulty exists in differentiating this condition from Crohn's disease because a similar endoscopic and histologic image can be seen. Fauconnier and Chapron [32] have described a relationship between specific types of pelvic pain symptoms, characteristics of the lesions, and semiology of the painful symptoms. Painful symptoms connected with DIE may present particular characteristics which distinguish them from painful symptoms of other origins. The painful semiology was found to be specific for anatomical location and for the organ affected: dyspareunia was associated with involvement of the uterosacral ligaments, painful defecation during menses with involvement of the posterior area, noncyclic pelvic pain and functional bowel signs with bowel involvement, and functional urinary tract signs with involvement of the bladder. Painful defecation during menses and severe dyspareunia were specifically connected to DIE infiltration of the pelvic nerves. In most cases the pain is provoked or aggravated by mobilization of the organs affected by the DIE lesions. The relationship between the severity of dysmenorrhea in women with posterior DIE and the indicators of the extent of the disease was evaluated by Chapron et al. [33]. The presence of a rectal or vaginal infiltration and extensiveness of adnexal adhesion has been shown to correlate with the severity of dysmenorrhea. The combination of endometrioma and pain is significantly related to the simultaneous presence of DIE [34]. Furthermore, another study by Chapron et al. [35] found that the mean number of DIE lesions was significantly higher and more severe in patients presenting an associated ovarian endometrioma. Thus, in a clinical context suggestive of DIE, when there is an ovarian endometrioma, the practitioner should investigate the extent of the disease to check for severe and multifocal DIE lesions. The history of patients at the time of adolescence has revealed that some events or symptoms in early menstruation are statistically more frequently associated with a later surgical diagnosis of DIE. Patients with DIE have significantly more positive family history of endometriosis and more absenteeism from school during menstruation. The oral contraceptive pill use and duration of treatment are more frequent and longer in patients with DIE. There is a higher incidence of pill use for severe primary dysmenorrhea before 18 years of age in patients with DIE [36]. Patients with a history of surgery for endometriosis show an increased prevalence of deeply infiltrating endometriosis. Furthermore, surgical history for endometriosis correlates significantly with number and severity of deeply infiltrating endometriosis lesions especially in the case of intestinal lesions [37]. Although solid data linking symptoms to size and localization of deep endometriosis are lacking, clinical symptoms remain crucial to suspect DIE, to use other diagnostic tools, and to decide on medical and/or surgical therapy [11]. Lafay Pillet et al. [38] developed a clinical model using the reproductive history and clinical symptoms of women with endometriomas to predict DIE based on clinical symptoms. Four variables were found to be independently associated with DIE: visual analogue scale of gastrointestinal symptoms ≥5 or of deep dyspareunia >5, duration of pain greater than 24 months, and severe dysmenorrhoea. A score <13 defined a low-risk group while a score ≥35 defined a high-risk group. In cases suggestive of DIE lesions, additional radiologic studies may help the skilled surgical team to identify and localize the deep lesions.

### 3.3. Diagnosis and Preoperative Work-Up of Intestinal Deep Infiltrating Endometriosis

Many exams can be used for the evaluation of bowel endometriosis; physical examination is helpful to detect the 50% of rectovaginal nodules >3 cm in diameter [26]. The aim of clinical and instrumental investigation is to (1) document the extent of the disease (2), plan a multidisciplinary treatment, and (3) counsel patients regarding the type of intervention and the possibility of intra- and postoperative complications.

Transvaginal ultrasonography is a routinary and noninvasive gynecologic exam that, according to a recent meta-analysis, can detect bowel endometriosis with pooled estimates of sensitivity and specificity of 91% and 98%, respectively [39]. By ultrasound evaluation, nodules appear as heterogeneous, hypoechoic, and more rarely speculated masses [40].

Barium enema examination and magnetic resonance imaging (MRI) are, with transvaginal ultrasonography, the gold standard for the noninvasive evaluation of bowel endometriosis, with or without involvement of the rectovaginal septum.

Barium enema is useful for assessing the extent of the disease; the radiological image of deep invasion of the bowel wall consists in an extrinsic mass compressing the lumen in association with the fine crenulation of the mucosa. Also bowel strictures, especially at the rectosigmoid junction, are characteristic of this disease. The limit of this diagnostic procedure is the impossibility of the exact evaluation of the distance to the anal sphincter [41]. MRI is useful for the diagnosis of multifocal endometriotic nodules and to define anatomical relationships, with a sensitivity and a specificity around 90%. MRI shows contrast enhanced mass or hyperintense foci on T1-weighted or fat-suppression T1-weighted images that are specific for hemorrhagic foci or hyperintense cavities secondary to endometriosis. On T2-weighted images nodules can be seen as hypointense masses with the signal of the tissue close to that of pelvic muscles [42, 43]. Rectosigmoidoscopy or colonoscopy are rarely used in clinical practice because endometriosis is an extrinsic and typically nontransmural disease [39, 41].

### 3.4. Medical and Surgical Treatment of Intestinal Deep Infiltrating Endometriosis

Treatment of intestinal DIE is difficult and challenging. Medical management of DIE with colorectal extension (with nonsteroidal anti-inflammatory drugs, oral contraceptives, gestagens, antigestagens, or GnRH agonists) is based on suppression of the symptoms, is not curative, and is often associated with significant side effects [14]. Nevertheless it is not clear if the medical management approach prevents disease progression, especially in more severe cases of endometriosis with colorectal extension. In addition, discontinuation of this therapy commonly results in recurrence [44]. Therefore, it is widely agreed that surgical management is the primary treatment for more severe forms of endometriosis, such as symptomatic DIE with colorectal extension [45, 46]. There is no consistent evidence in the literature to determine whether medical preoperative treatment is associated with a significant benefit; nevertheless costs and side effects of these therapies should be considered [47]. Some authors suggest that preoperative danazol treatment could be useful to increase the pregnancy rate [48].

To diagnose and uniformly classify bowel endometriosis, exploratory laparoscopy is necessary. The above-mentioned imaging techniques are noninvasive and helpful to confirm the clinical suspicion and to assess the extent of the disease. Usually a gynecologist expert in endoscopic surgery perform the exploratory laparoscopy; in our opinion the cooperation with a colorectal surgeon is recommended. The surgeon has to look for the presence of suspicious lesions in the uterus, uterosacral ligaments, pelvic peritoneum, ovaries and ureters, sigmoid colon, and the upper rectum. An extraperitoneal surgical approach is sometimes necessary to explore the pouch of Douglas because it is often obliterated by perilesional adhesions [9, 49]. The aim of the surgery is to obtain pain relief, prevent recurrence rates, and improve fertility; it is also important to prevent the formation of postoperative adhesions. To achieve these results a total removal of endometrial implants without compromising ovarian function is mandatory.

The therapeutic strategy should not be influenced by the association of different types of endometriotic lesions, such as endometriomas, peritoneal endometriosis, or DIE; the complete excision of all implants, saving normal tissue, is of paramount importance [50, 51].

Nevertheless, more than 70% of women with DIE still underwent segmental bowel resection [52].

Several surgical procedures for endometriosis with bowel involvement have been described using a laparoscopic, a laparotomic, a transvaginal, or a combined approach.

Laparoscopy is preferred to open surgery since it is usually associated with a better and shorter postoperative recovery and a better cosmetic result [53]; both procedures are equally safe and effective in the treatment of endometriosis. As it is usually hoped for an oncologic disease, also for the treatment of deep endometriosis, it is recommended to refer the patient to an expert center that offers a minimally invasive treatment in a multidisciplinary context [54].

Laparoscopic surgical procedures for rectosigmoid DIE lesions can have a conservative or a radical purpose. Nodulectomy is used for a conservative approach and can be performed using several techniques: traditional or reversal rectal shaving (defined as superficial peeling of bowel serosal and subserosal endometriosis with diathermy loop or laser), mucosal skinning, full-thickness anterior rectal wall excision (defined as selective excision of the bowel endometriotic lesion without opening of the bowel wall), and full-thickness disc excision (defined as selective excision of the bowel endometriotic lesion, followed by closure of the bowel wall). The aim of these approaches is strictly intended to remove localized endometriosis nodules. Radical surgery consists in segmental bowel resection of the affect tract, followed by primary colorectal anastomosis with or without protective ileostomy (depending on the distance between DIE localization and anal sphincter).

The preoperative imaging examinations of intestinal DIE lesions should contain information of fundamental importance for the planning of procedure, such as size of the lesion, depth of infiltration, distance from anal verge, and multicentricity and multifocality of the lesions. The size of the nodule and the percentage of bowel circumference involved by the DIE lesion can be similarly evaluated by MRI and TVUS. In general, only patients with intestinal DIE lesions measuring up to 25–30 mm may be candidates for conservative surgery, either shaving rectal/mucosal skinning or disk resection [17]. The depth of infiltration of endometriotic lesions into the bowel wall is another important variable to consider in the surgical treatment of choice. In this context, a distinction can be drawn between the presence of endometriotic lesions on the bowel serosa and endometriotic lesions infiltrating the muscularis. Lesions of the serosa without infiltration do not justify any specific bowel procedure from a surgical point of view. This superficial form of serosal bowel endometriosis may be treated by surgical shaving or eventually by full-thickness discoid excision if shaving resulted in significant bowel trauma [52].

In general, classical shaving should be indicated for superficially DIE lesions affecting the intestinal wall no deeper than the muscular layer, preserving the mucosa layer. Full-thickness anterior rectal wall excision or discoidal resection are appropriate options when there is evidence of singular endometriotic nodule, smaller than 30 mm, infiltrating intestinal wall deeper than the muscular layer and affecting less than 1/3 of the intestinal circumference. Segmental bowel resection is appropriate when DIE lesion is bigger than 30 mm [52].

The distance of the DIE lesion from the anal verge is important for surgical planning. This distance can be determined by TVUS or pelvic/abdominal MRI with greater accuracy. An independent risk factor for the occurrence of anastomotic leaks after intestinal segmental resection is the colorectal anastomosis being less than 10 cm away from the anal verge; therefore, it is advisable to consider a temporary protective ileostomy in these cases [17]. Finally an important characteristic that should be taken into account before deciding on the surgical strategy for DIE is multifocality and multicentric involvement. In almost 70% of the cases, intestinal endometriosis lesions are associated with DIE in other locations, justifying specific associated surgical procedures for the uterosacral ligaments, vagina, bladder, and/or ureter. In the presence of multifocal or multicentric lesions, the option of surgery is usually restricted to intestinal resection in order to obtain the complete treatment of the disease [52]. Owing to the paucity of comparative studies [29, 55], it should be emphasized that the present available data are provided by retrospective series reported by surgeons who generally perform only one technique. Consequently, it is unclear from the literature when to use which procedure, and there are no available objective criteria to indicate the use of one procedure rather than the other, so surgical management is often based on little evidence and tends to reflect the personal convictions and experience of surgeons [29, 55, 56].

To summarize, when the intestinal tract is involved, a multidisciplinary approach has been proposed as mandatory [57, 58], since best results in terms of improvement of symptoms and quality of life can be guaranteed by a complete surgical excision of all endometriotic implants [29, 52–59]. Deep infiltrating endometriosis is a global pathology that may involve different structures. A multidisciplinary approach should be recommended to achieve appropriate disease management. Collaboration between gynaecologists, urologists, and colorectal surgeons enabled a successful management of the case in one surgical intervention providing minor risk of complications, shorter hospital stay, and faster functional recovery [60, 61].

### 3.5. Postoperative Care of Intestinal DIE

Antibiotics should be administered as one shot when the intestinal wall has not been opened, whereas full-thickness resection requires 7 days of antibiotic treatment. Following a muscularis defect and single-layer suture, or full-thickness resection and double-layer suture, the patient remains nil by mouth for 4 and 7 days, respectively. Postoperative care after surgery requires strict follow-up with early repeat laparoscopy to immediately treat any complications, including bleeding, infection, late ureteral or bowel perforation, or fistulae. When a complication occurs more than two weeks after surgery, the risks and advantages of immediate intervention should be discussed considering the patient's clinical condition [11, 62].

### 3.6. Complications and Recurrence of the Disease after DIE Surgery

Conservative procedure demonstrates low rate in morbidity and urinary/intestinal postoperative complications compared to a radical approach. Nevertheless, on the one hand, sometimes complete resectability is not totally achieved by conservative approach due to the presence of microscopic residual endometriosis close to margin of resection, which increases the recurrence risk [63]. On the other hand, colorectal resections have good results in long-term pain relief and fertility. The more common complication of the radical treatment of intestinal DIE is anastomotic leakage followed by rectovaginal fistula. Anastomotic leakage occurs more frequently when anastomosis is performed close to the anal sphincter. Protective ileostomy can help to reduce this complication [64, 65]. Postoperative rectovaginal fistula occurs more frequently when both rectum and vagina are opened during the procedure. In this case, a two-stage approach with vaginal surgery followed by colorectal resection can reduce the risk of fistula [62]. Recurrence is a possible complication after laparoscopic segmental bowel resection and occurs in about 20% of cases [66]. Some authors describe a higher recurrence rate for conservative management compared to a radical approach (17.6% and 5.8%, resp.), while other authors did not show any significant difference [67, 68]. Several factors may play role in recurrence rate: accumulating evidence suggests that positive bowel resection margins, obesity, and age are significantly associated with endometriosis recurrence. Wide margin of excision, independently from the type of surgical approach, seems to be the greatest factor capable of decreasing the recurrence rate [22]. Lesion's characteristics should guide surgeons regarding the surgical choice technique: discoid bowel resection should be preferred when maximum diameter does not exceed 3 cm with a maximum bowel circumference involvement of 50% [55]. Histological features can also provide information concerning possible rate recurrence: multicentric bowel involvement, characterized by deep nodules with surrounding fibrosis, reduces the probability of radical excision and consequently increases the recurrence rate [24, 25]. As it widely accepted, the extension and localization of the disease can play a pivotal role in the arising and exacerbating of chronic pelvic pain [4, 69] and related decrease in quality of life [70]. In addition, accumulating evidence suggests that the interaction between immune system and endometriotic cells may cause a breakdown of the peritoneal immune surveillance [71, 72], resulting in a diminished apoptosis of endometriotic cells [73, 74] and disturbance of epigenetic expression of several genes of paramount importance for the progression of the disease [75, 76].

## 4. Conclusions

The debate on what is the standard of care in surgical treatment of intestinal deep infiltrating endometriosis is not completely clarified. According to our data analysis, universally accepted points are a standardized preoperative assessment for bowel endometriosis' diagnosis, an adequate patient counseling, and a multidisciplinary minimally invasive surgical approach in an expert center. A patient-tailored approach is required and the less invasive radical option should be chosen.

Laparoscopy is preferable to laparotomy, as it decreases postoperative discomfort, operative morbidity, length and costs of hospitalization, improve cosmetic healing, and facilitate return to normal function. A colorectal surgeon expert on intestinal endometriosis should make a correct decision regarding whether to perform a full-thickness excision, a shaving, or a bowel resection.

Taking advantage of the ongoing evolution of minimally invasive approaches, future investigations should be focused on ensuring the radical excision of endometriotic lesions saving the function of all the organs involved by the disease, using minimal surgical access possible. Future efforts should improve long-term outcomes with regard to symptoms, quality of life, cosmetic outcome, recurrence rate and fertility.
